# MicroRNA-150-5p inhibits the proliferation and invasion of human larynx epidermiod cancer cells though regulating peptidyl-prolyl cis/trans isomerase

**DOI:** 10.1016/j.bjorl.2023.03.003

**Published:** 2023-03-13

**Authors:** Hailin Chen, Xuwei Cai, Baowen Du, Jianpiao Cai, Zhengzheng Luo

**Affiliations:** Huizhou Municipal Central Hospital, ENT and HN Surgery Department, Huizhou, China

**Keywords:** MicroRNA-150-5p (miR-150-5p), Peptidyl-prolyl cis/trans isomerase NIMA-interacting 1(PIN1), Laryngeal squamous cell carcinoma (LSCC), Proliferation, Invasion

## Abstract

**Objective:**

This study aimed to investigate the molecular mechanism of miR-150-5p regulating the malignant biological behavior of Human Epidermoid cancer cell (HEp-2) by targeting peptidyl-prolyl cis/trans isomerase NIMA-Interacting-1 (PIN1).

**Methods:**

Firstly, qRT-PCR and Western blot were adopted to detect the expression levels of miR-150-5p and PIN1 in cancer tissue and paracancerous tissues of patients with LSCC, and those in human bronchial epithelial cells (16 HBE) and HEp-2. Next, the targeted relationship between miR-150-5p and PIN1 was assessed by bioinformatics website and dual-luciferase reporter assay, followed by their correlation analysis. Besides, after interfering with miR-150-5p or PIN1 expression in HEp-2 cells, CCK-8, cell colony formation assay, and transwell assay were utilized to detect the proliferation, viability, and invasion of cells, respectively. Subsequently, the protein levels of MMP-2, MMP-9, and EMT-related proteins in HEp-2 cells were checked by Western blot.

**Results:**

Expression of miR-150-5p was down-regulated in LSCC tissues and HEp-2 cells. Moreover, miR-150-5p suppressed proliferation and invasion of HEp-2 cells, affected protein expression related to MMP and EMT, thereby inhibiting development of cancer. The expression of PIN1 was significantly increased in cancer tissues and HEp-2 cells, and there was a targeted relationship and negative correlation between miR-150-5p and PIN1 in cancer tissue. However, overexpression of PIN1 could reverse the effect of miR-150-5p on the proliferation and invasion of HEp-2 cells.

**Conclusion:**

In a nutshell, there is a targeted relationship between PIN1 and miR-150-5p. Besides, miR-150-5p can inhibit the proliferation and invasion of HEp-2 cells by regulating the expression of PIN1.

**Level of evidence:**

3.

## Highlights

• MiR-150-5p is down-regulated in Laryngeal Squamous Cell Carcinoma (LSCC).
• PIN1 is a target gene of miR-150-5p.
• MiR-150-5p inhibits the proliferation and invasion of LSCC cells by targeting PIN1.

## Introduction

Laryngeal carcinoma is a prevalent malignancy of the head and neck, accounting for 0.5%–1% of new cases or death of systemic tumors.^1^ The main pathological type of laryngeal carcinoma is squamous cell carcinoma. In addition, it’s reported that the occurrence of laryngeal carcinoma is associated with smoking, alcohol consumption, environmental factors, radiation, viral infections and deficiency of trace elements.^2^ Related data displayed that laryngeal carcinoma is becoming more common in China and even the whole world,^3^ not only seriously threatening human life and health, but also affecting their quality of life. Although new therapies such as surgery, chemotherapy, and radiotherapy can improve the clinical efficacy of laryngeal cancer, the efficacy for patients with advanced laryngeal cancer is unsatisfactory, with a five-year survival rate of 30%–40%.^4^, ^5^ Recently, targeted therapy has shown some potential in the management of laryngeal cancer. Many biomarkers have been identified as being involved in the growth of Laryngeal Squamous Cell Carcinoma (LSCC). A study pointed out that, due to important role in centrosome replication, Peptidyl-prolyl cis/trans isomerase NIMA-Interacting 1 (PIN1) is highly expressed in the vast majority of human tumors and participates in the control of the cell cycle.^6^ Similarly, another report claimed that PIN1 may serve as a viable therapeutic target of LSCC because it is not only highly expressed in LSCC but also linked to the risk of developing cancer.^7^ However, the mechanism of PIN1 changes has not yet been clarified, and further exploration is still required.

MicroRNAs (miRNAs), a group of endogenous non-coding RNAs of 18–25 nucleotides in length, regulates gene expression by specifically binding to the 3′-UTR of target genes.^8^, ^9^ A number of biological activities, like cell differentiation, proliferation, invasion, metastasis, and apoptosis, are regulated by miRNAs as well.^10^ Quite a few studies stated that miRNAs, including microRNA-150-5p (miR-150-5p), affect the occurrence and progression of laryngeal carcinoma by either promoting or inhibiting it.^11^ Through RNA sequencing, Koshizuka et al. discovered that this RNA was considerably under expressed in Head and Neck Squamous Cell Carcinoma (HNSCC) and linked to a worse survival in HNSCC patients.^12^ Additionally, some researchers have discovered that miR-150-3p and miR-150-5p can inhibit SPOCK1 expression in HNSCC cells and reduce the invasion of cancer cells.^13^ Moreover, Li et al. proposed that miR-150-5p expression was markedly curtailed in oral squamous cell carcinoma cells and was associated with increased cancer cell malignancy.^14^ In brief, miR-150-5p is critical for the growth of HNSCC. However, it is yet unknown how this RNA affects LSCC. Interestingly, bioinformatics analysis revealed PIN1 as a possible target of this RNA, but their correlation needs further validation. Therefore, the objective of this article was to investigate the regulatory effect of miR-150-5p on PIN1 and clarify the mechanism of miR-150-5p affecting the proliferation and invasion of laryngeal cancer cells. Apart from that, this paper also aimed to provide clues and basis for the exploration of potential biomarkers of the diagnosis, treatment, and prognostic detection of laryngeal cancer.

## Methods

### Sample collection

Cancer tissue (LSCC group, n = 8) and paracancerous tissue (Normal group, n = 8) were extracted from LSCC patients admitted to our hospital. Besides, all patients offered the written informed consent forms, and the Ethics Committee of our hospital gave its approval to this study.

### Cell culture

Human Bronchial Epithelial cells (16 HBE) and Human Epithelial cancer cells (HEp-2) were offered by American Type Culture Collection (USA). Next, DMEM medium (Gibco, USA) with 10% Fetal Bovine Serum (FBS) (Hyclone, USA) was adopted for 16 HBE cell culture, while 1640 medium (Gibco, USA) with 10% FBS (Hyclone, USA) for HEp-2 cell culture. All culture media were added with 1% 100 U/mL penicillin ‒ 0.1 mg/mL streptomycin (Beyotime, China) and cultured in a sterile incubator at 37 °C with 5% CO_2_.

### Cell transfection

HEp-2 cells were cultured to the logarithmic phase, followed by digestion and passage. Next, HEp-2 cells in the logarithmic phase were gathered, diluted to 2 × 10^6^ cells/mL, and inoculated into 6-well plates for culture. Subsequent to the fusion degree of cells reached 80%–90%, the culture was discontinued, and the transfection was performed. Following the operating instructions of Lipofectamine™ 2000, miR-150-5p mimics, NC mimics, miR-150-5p inhibitor, NC inhibitor (GenePharma, China), vector, and PIN1 over-expression plasmid (ThermoFisher, USA) were transfected into HEp-2 cells using Lipo2000, respectively. Subsequently, the plates were developed for 24 h in a CO_2_ incubator at 37 °C. Finally, a light microscopy was applied to measure transfection efficiency.

### Real time quantitative reverse transcription-polymerase chain reaction (qRT-PCR)

Total RNA was obtained from the collected tissues and cells using Total RNA extraction kit (Takara, USA), and the miRNAs were extracted with miRNA extraction and isolation kit (Tiangen, China). Both extracted total RNA and miRNAs were stored at −80 °C. By reverse transcribing miRNA and mRAN with the directions on the reverse transcription PCR kit (Takara, USA) and the miRNA cDNA First Strand Synthesis Kit (Tiangen, China), the complementary DNA was synthesized. Checks were made on the complementary DNA that was created in terms of purity and concentration. Next, the Polymerase Chain Reaction (PCR) of mRAN was carried out based on the instruction of real-time PCR (Takara, USA). The reaction procedure went as follows: 95 °C for 30 s; 95 °C for 10 s; 57 °C for 20 s; 72 °C for 20 s, for 40 cycles. The PCR of miRNA was performed according to the miRNA fluorescence quantitative detection kit (Tiangen, China), and the reaction program went like this: 95 °C for 15 min; 94 °C for 20 s; 60 °C for 34 s, for 40 cycles. Taking GAPDH as an internal control, the 2^−ΔΔCt^ method was utilized to perform data analysis. The sequences of primers adopted were shown in Table 1.

**Table 1:** Primer sequences for qRT-PCR.

Genes	Primer sequences
PIN1	Forward: 5'-TTTGAAGACGCCTCGTTTGC-3'
	Reverse: 5'-GTGCGGAGGATGATGTGGAT-3'
miR-150-5p	Forward: 5′-TCTCCCAACCCTTGTACCAGTG-3′
	Reverse: 5′-GTGCGTGTCGTGGAGTC-3′
U6	Forward: 5′-GCTTCGGCAGCACATATACTAA-3′
	Reverse: 5′-AACGCTTCACGAATTTGCGT-3′
GAPDH	Forward: 5'-CGGAGTCAACGGATTTGGTCGTAT-3'
	Reverse: 5'-AGCCTTCTCCATGGTGGTGAAGAC-3'

### Detection of cell proliferation by cholecystokinin octapeptide (CCK-8)

The transfected HEp-2 cells were put into a 96-well plate with 2000 cells/well, and 100 μL fresh complete medium was replaced at an interval of 24 h. Subsequently, 10 μL Cholecystokinin Octapeptide (CCK-8) solution (Abcam, USA) and 90 μL fresh complete culture were introduced to each tested wells. Next, the 96-well plate was positioned in an incubator and allowed to culture for a full hour. Lastly, molecular devices were employed to monitor the absorbance of 450 nm wavelength.

### Colony formation assay

The transfected HEp-2 cells seeded in a 6-well plate were subjected to 2–3 weeks of static culture at 37 °C in an incubator containing 5% CO_2_. Upon macroscopic colony formation, the cell culture was discontinued. To count the number of colonies, the supernatant was aspirated, cells were fixed with methanol at ambient temperature for 30 min, and the staining was performed for 30 min with 0.5% crystalviolet (Sigma-Aldrich, USA). Later, the number of cell clones larger than 50 were counted under a microscope to determine the colony formation rate. The formula was displayed as follows: colony formation rate (%) = (number of colonies/number of the seeded cells) ×100%.

### Detection of cell invasion by transwell assay

Matrigel (BD, Franklin Lakes, USA) was removed from −20 °C, left at 4 °C overnight to become liquid, and diluted in a ratio of Matrigel: 4 °C serum-free mediu = 6:1. Next, 100 μL diluted matrigel was supplemented to the upper transwell chamber at 37 °C for 3–5 h to turn it into solid. Then, the lower chamber received 500 μL of 10% FBS medium, whereas the upper chamber received 100 μL of cell suspension. The migrated cells were fixed with methanol for 30 min following a 24 h development. Upon removal of non-migrated cells, the staining was employed with 0.5% crystal violet for 30 min. Finally, cells were counted under a microscope.

### Dual luciferase reporter assay

Transfection was conducted once HEp-2 cells had fused to an extent of 80%–90%. Shortly speaking, miR-212-3pmimics were co-transfected into HEp-2 cells along with the constructed Mutant (PIN1-MUT) and Wild-Type (PIN1-WT) dual luciferase reporter vectors of PIN1 (ThermoFisher, USA). After transfection, the HEp-2 cell culture was performed for 48 h then collected for 20 min of lysis at ambient temperature. Next, the centrifugation was carried out and the supernatant was collected. Direct addition of the luciferase substrate was then made, followed by a measurement of the amount of luciferase activity by luminescence instrument. Later, relative firefly luciferase activity was calculated with Renilla luciferase activity serving as an internal control.

### Western blot

The tissues and cells of each group were lysed 30 min with RIPA lysis buffer, and then crushed by sonication in ice bath. Next, the protein concentration was measured once the proteins had been collected. The proteins were transferred to PVDF membrane after Sodium Dodecyl Sulphate-Polyacrylamide Gel Electrophoresis (SDS-PAGE) and sealed at ambient temperature for 1 h. Later, primary antibodies (Proteintech, USA): E-cadherin (Cat nº 20874-1-AP), N-cadherin (Cat nº 22018-1-AP), MMP-9(Cat nº 10375-2-AP), Vimentin (Cat nº 10366-1-AP), Matrix Metalloproteinase (MMP)-2 (Cat nº 10373-2-AP), PIN1 (Cat nº 10495-1-AP) were added respectively, then the incubation was conducted overnight at 4 °C. Washed twice with PBS, the membrane was supplemented with the diluted enzyme-labeled second antibody for 1 h incubation at indoor temperature. The protein level was determined with β-actin being an internal control.

### Statistical analysis

Using the SPSS 26.0 program, statistical analysis was performed on the experimental data. When comparing two groups, the *t*-test was adopted, and when comparing multiple groups, one-way analysis of variance. The results were provided as the Mean ± Standard Deviation (Mean ± SD), and *p* < 0.05 represented a significant difference.

## Results

### Down-regulation of miR-150-5p in laryngeal squamous cell carcinoma tissues and HEp-2 cells

**Figure 1:**
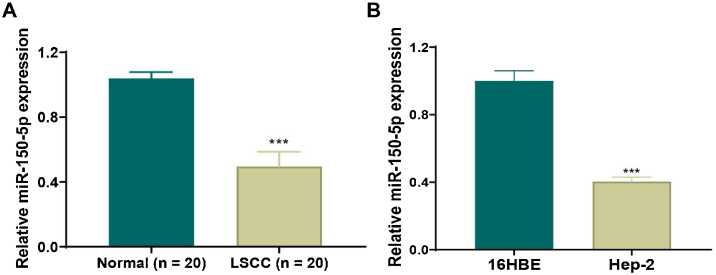
Down-regulated expression of miR-150-5p in laryngeal squamous cell carcinoma. (A) The expression of miR-150-5p in laryngeal squamous cell carcinoma and paracancerous tissues was detected by qRT-PCR; (B) The expression of miR-150-5p in normal Human Bronchial Epithelial cells (16 HBE) and Human Epithelial cancer cells (HEp-2) derived from a larynx carcinoma was tested by qRT-PCR; ***p < 0.001.

To clarify the role of miR-150-5p in LSCC, qRT-PCR was applied to observe its level in LSCC and normal tissues. We discovered that this RNA expression level was significantly reduced in LSCC tissue (Fig. 1A). Also, there was the same trend of expression at the cellular level. The expression of this RNA notably descended in HEp-2 cells relative to normal 16 HBE cells (Fig. 1B). In a word, reduced expression of this RNA may be related to the progression of LSCC.

### MiR-150-5p can inhibit the proliferation and invasion of HEp-2 cells

**Figure 2:**
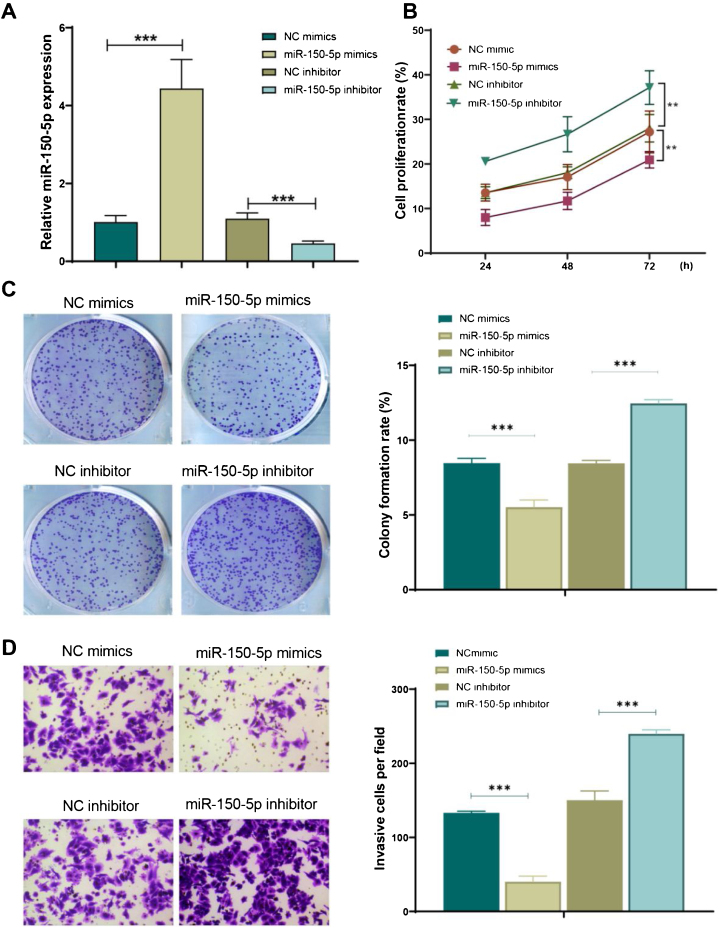
MiR-150-5p inhibits the proliferation and viability of HEp-2 cells. (A) qRT-PCR to detect the expression level of miR-150-5p in each group of HEp-2 cells; (B) CCK-8 method to check the cell proliferation rate of cells; (C) Cell colony formation assay to observe the cell proliferation of each group; (D) Transwell assay to test the invasion ability of HEp-2 cells in each group. ***p < 0.001.

Subsequently, for further observing the function of miR-150-5p in the development of LSCC, its expression level was under expressed (miR-150-5p inhibitor) or overexpressed (miR-150-5p mimics) in HEp-2 cells. The outcomes of qRT-PCR indicated that miR-150-5p mimics could remarkably enhance the expression level of this RNA in HEp-2 cells (*p* < 0.01), however, inhibitor of this RNA significantly declined it (*p* < 0.01) (Fig. 2A). Therefore, a successful transfection was obtained. Later, the cell proliferation of each group was observed with the help of cell colony formation assay and CCK-8 assay. The outcomes suggested that the miR-150-5p mimics group showed a markedly decreased cell proliferation rate (*p* < 0.01); however, the rate visibly climbed after inhibition of this RNA (*p* < 0.01) (Fig. 2B‒C). Furthermore, the invasion of cells in each group was observed by transwell assay. The findings exhibited that the invasion of cells was observably reduced after the overexpression of this RNA (*p* < 0.01) but enhanced after its inhibition (*p* < 0.01) (Fig. 2D). In brief, this RNA can prevent HEp-2 cells from proliferating and invasively spreading.

### MiR-150-5p can inhibit matrix metalloproteinase and the progression of epithelial-mesenchymal transition

**Figure 3:**
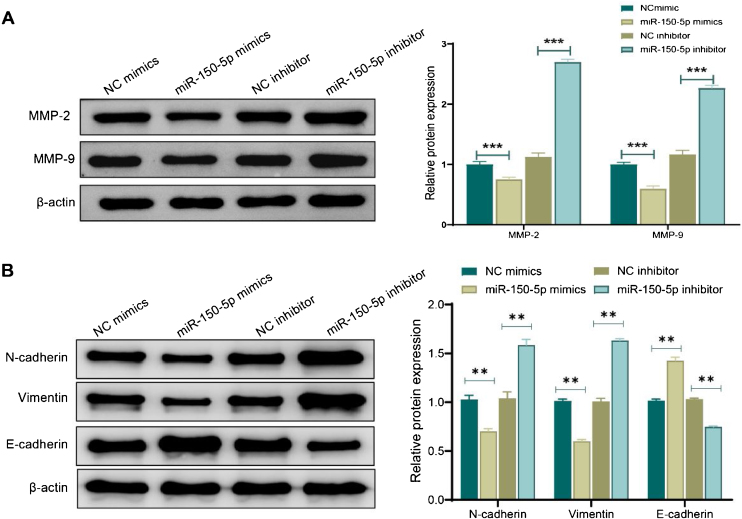
Effect of miR-150-5p on the expression of Matrix Metalloproteinase (MMP) and Epithelial-Mesenchymal Transition (EMT)-related proteins in Hep-2 cells. (A‒B) Western blot to assess the expression of Matrix Metalloproteinase (MMP)-related proteins MMP-2 and MMP-9 (A) and Epithelial-Mesenchymal Transition (EMT)-related proteins (N-cadherin, Vimentin and E- cadherin) (B) in Hep-2 cells; **p < 0.01; ***p < 0.001.

Western blot analysis was performed to evaluate the expression of MMP and proteins associated with the Epithelial-Mesenchymal Transition (EMT) for further investigating the mechanism by which miR-150-5p prevents the invasion of HEp-2 cells. Shortly speaking, in the miR-150-5p mimics group, protein expression levels of MMP-2, MMP-9, Vimentin and N-cadherin in cells were notably down-regulated (*p* < 0.01) while the E- cadherin expression was markedly raised (*p* < 0.01). Nevertheless, miR-150-5p inhibitor group displayed a significant increase in the expression levels of MMP-2, MMP-9, Vimentin and N-cadherin (*p* < 0.01) while a notable reduction in the E-cadherin expression (*p* < 0.01) (Fig. 3A‒B). All in all, this RNA could not only suppress critical protein expression of MMPs but also inhibit the invasion of HEp-2 by inhibiting the EMT process.

### MiR-150-5p can targetedly suppress the expression of PIN1

**Figure 4:**
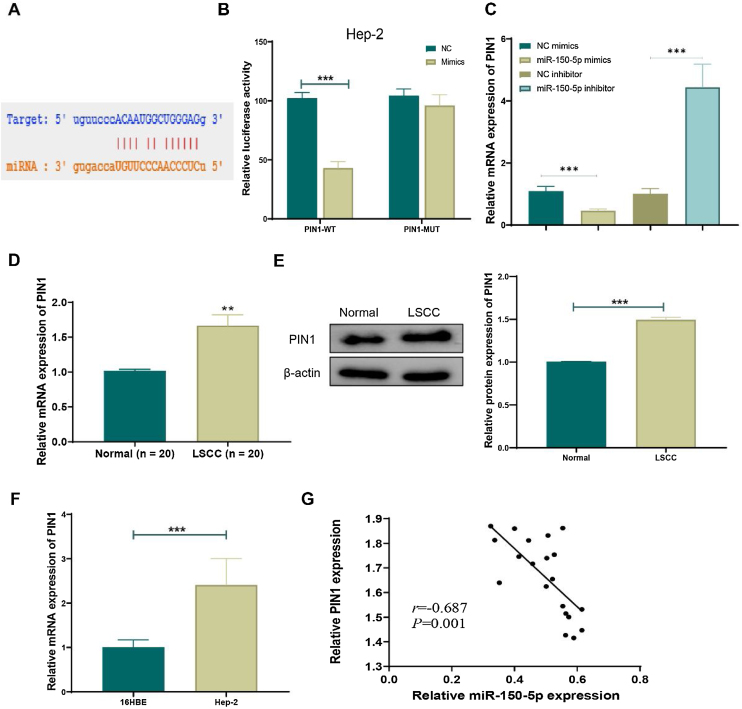
MiR-150-5p targeted inhibits PIN1 expression. (A) Potential target gene sequences of miR-150-5p were predicted by the online prediction network TargetScan; (B) Dual-luciferase reporter assay to verify the targeted relationship between miR-150-5p and PIN1; (C) QRT-PCR to detect PIN1 expression in cells with different miR-150-5p expression levels; (D) Expression of PIN1 detected by qRT-PCR in tissues from LSCC and Normal groups; (E) Western blot to meaure PIN1 protein expression in LSCC and Normal groups; (F) QRT-PCR for testing PIN1 expression in 16 HBE and Hep-2 cells; (G) Pearson correlation analysis for relation between miR-150-5p and PIN1 expression in laryngeal squamous cell carcinoma tissues (n = 8); **p < 0.01, ***p < 0.001.

TargetScan (http://www.targetscan.org/vert_72/), an online prediction network for miRNA target genes, was employed to predict target genes of miR-150-5p in order to reveal its mechanism. The prediction outcomes suggested an interaction between PIN1 and this RNA (Fig. 4A). Next, dual-luciferase reporter assays further uncovered that overexpression of this RNA noticeably inhibited luciferase activity of PIN1-WT (*p* < 0.01) but didn’t affect luciferase activity of PIN1-MUT (Fig. 4B). Additionally, qRT-PCR results exhibited that the overexpression of this RNA obviously lowered PIN1 expression, whereas its knockdown greatly increased PIN1 expression in cells (Fig. 4C). Moreover, the observation results of clinical tissues displayed that, relative to the Normal group, both protein and mRNA expression of PIN1 were significantly elevated in the LSCC group (Fig. 4D‒E). Similarly, PIN1 expression was notably increased in HEp-2 cells relative to that in 16 HBE cells (Fig. 4F). Furthermore, the Pearson correlation coefficient analysis disclosed the negative correlation between the expression of PIN1 and this RNA (Fig. 4G). Hence, this RNA could targetedly suppress PIN1 expression in LSCC.

### Overexpression of PIN1 can reverse the effect of miR-150-5p on the proliferation and invasion of HEp-2 cells

**Figure 5:**
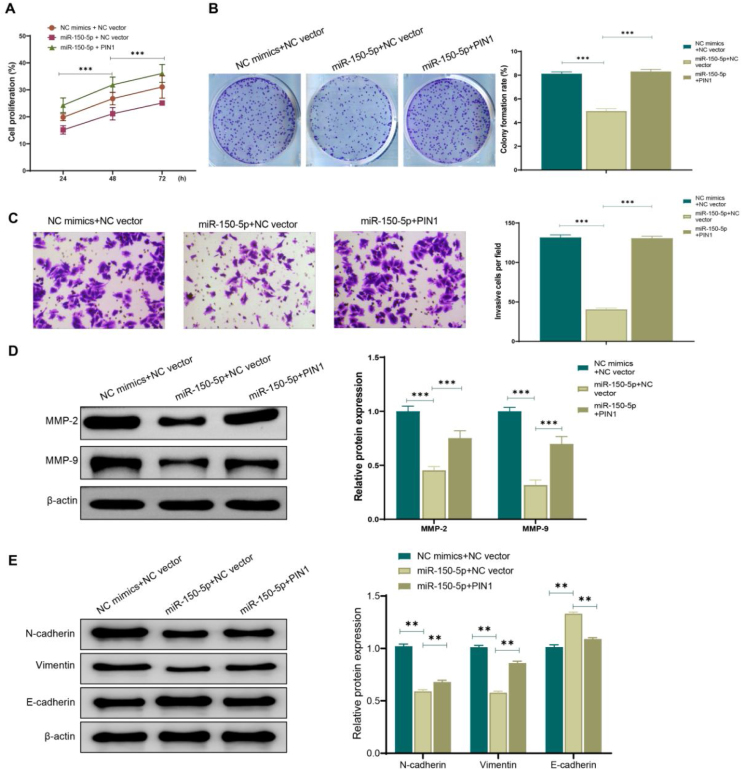
Overexpression of PIN1 promotes proliferation and invasion of laryngeal cancer cells. (A) CCK-8 assay for detecting the cell proliferation rate of HEp-2 cells in each group; (B) Colony formation assay for observing the cell proliferation of Hep-2 cells in each group; (C) Transwell assay for checking the cell invasion of HEp-2 cells; (D‒E) Western blot for assessing the expression of Matrix Metalloproteinase (MMP)-related proteins MMP-2 and MMP-9 (D) and Epithelial-Mesenchymal Transition (EMT)-related proteins N-cadherin, Vimentin and E- cadherin (E) in HEp-2 cells. **p < 0.01, ***p < 0.001.

To further confirm the role of the association between miR-150-5p and PIN1 in HEp-2 cells, this RNA and PIN1 was simultaneously highly expressed in cells. According to the outcomes of transwell assay, colony formation assay and CCK-8 assay, miR-150-5p (miR-150-5p + NC vector) overexpression effectively weakened the ability of HEp-2 cells to proliferate and invasively spread; while on this basis, PIN1 (miR-150-5p + PIN1) overexpression could relieve the inhibition of this RNA on the proliferation and invasion of HEp-2 cells (Fig. 5A–C). Additionally, western blot results also presented that PIN1 (miR-150-5p + PIN1) overexpression could reverse the impact of this RNA on the MMP and EMT-related protein expression levels in HEp-2 cells (Fig. 5D‒E). It makes sense to assume that the inhibition of laryngeal cancer cell growth and invasion by miR-150-5p is likely reversed by PIN1 overexpression.

## Discussion

LSCC is one of the most prevalent head and neck malignancies in humans with high morbidity and mortality, and a poor prognosis.^15^, ^16^ Despite recent advancements in the diagnosis and management of laryngeal cancer, the five-year survival rate has hardly improved over the previous 20 years. Early diagnosis of early lesions in high-risk groups may contribute to improving survival.^17^ Therefore, the search for suitable biomarkers is vital for the diagnosis and prognosis of LSCC. MiRNAs are a crucial part of cell signaling pathways and have been shown to be essential regulators of a variety of biological activities, like angiogenesis, stress response, proliferation, differentiation, and apoptosis of cells.^18^ Specifically, miRNAs function by binding the 3′-UTR region of multiple target genes. Dysregulated expression of miRNAs has been found to encourage the occurrence and progression of cancer.^19^ At present, there are no studies on the association between this RNA and LSCC. Our paper examined the effect of this RNA on HEp-2 cells in LSCC tissues, excavated its targets, and explored the related mechanism of action for the first time. Recent research has demonstrated that this RNA can be implicated in the emergence of a number of malignancies. For instance, it’s reported that this RNA inhibits oncogenes, like ovarian cancer, cervical cancer, and colorectal cancer.^20^ However, some investigations have also demonstrated that this RNA is a pro-oncogene. For example, Inoue et al. discovered that stomach cancer tissues had considerably increased level of this RNA.^21^ Moreover, Li et al. believed that knockdown of this RNA could greatly weaken the proliferation ability of multiple myeloma cells.^22^ Overall, there is some controversy regarding the function of this RNA in cancer development. Such controversy may be caused by the diversity of the functions of miRNAs, and the diversity is particularly common in cancer. In this study, miR-150-5p expression was lowered in both laryngeal carcinoma cell lines and LSCC tissues. Subsequently, for clearing the function of this RNA in LSCC, overexpression or knockdown of this RNA was performed in HEp-2 cells. The outcomes of cell invasion assay, colony formation assay, and CCK-8 assay indicated that overexpression of this RNA obviously prevented HEp-2 cell from proliferating and invading. In a word, this RNA functions as a tumor suppressor gene in LSCC.

In many studies, the tumor proliferation and invasion have been linked to the remodeling of Extracellular Matrix (ECM) through proteases such as MMPs.^23^ MMPs are a class of zinc-containing proteases that can degrade ECM. Through disrupting the degradation balance of the matrix, MMPs can promote cancer cells to break the chemical barrier constructed by Basement Membrane (BM) and ECM, thereby invading surrounding tissues and metastasize to distant tissues. Furthermore, MMPs have been confirmed to promote EMT and serve as one of the key factors in cancer metastasis.^24^, ^25^ The ECM and BM are degraded as a result of MMP2/9 overproduction or enhanced activity, which also encourages tumor cell invasion and metastasis. Therefore, down-regulation of MMP2 and MMP9 expression is important in inhibiting the invasion and metastasis of tumors.^26^ EMT indicates the changes in the plasticity of epithelial cells, the loss of intercellular adhesion, the remodeling of ECM, and the acquisition of mesenchymal cell characteristics.^27^ The EMT process (up-regulation of mesenchymal cell markers Vimentin and N-cadherin, loss of epithelial cell marker E-cadherin) allows cancer cells to migrate and invade more readily. Research have claimed that EMT are essential for the occurrence, invasion and metastasis of malignant tumors, and the invasion progression of malignant tumors can be relieved by inhibiting the EMT process.^28^ In this article, overexpression of this RNA could obviously lower the MMP-2, MMP-9, Vimentin and N-cadherin expression while enhance the E-cadherin expression. Such outcome in this study suggested that this RNA could reduce the degradation of ECM by suppressing the MMP expression and inhibit the HEp-2 cells to proliferate and invade via suppressing the EMT process.

Previous research has pointed out that miRNAs can affect the expression of target genes via binding to the 3’-UTR of mRNAs. Actually, a great many reports have discovered target genes of the miR-150-5p. For instance, this RNA can targeted regulate CPSF4 in colorectal cancer^29^ and targeted impact CCR2 in breast cancer.^30^ In this paper, bioinformatics software such as TargetScan was applied to dig out the mechanism of this RNA in LSCC. According to the results, binding sites of this RNA were predicted to exist in the 3’-UTR of PIN1 mRNA. Moreover, results from dual-luciferase reporter assay verified PIN1 as a target gene of this RNA. Unsurprisingly, PIN1 expression was elevated in clinical LSCC tissues and cell lines and negatively correlated with expression of this RNA. PIN1 has already been considered a cancer-promoting factor.^31^ Up-regulation of PIN1 in pancreatic cancer has been reported to activate NF-κB -IL-18 and stimulate the proliferation and invasion of cancer cells.^32^ By activating Wnt/β-catenin signaling, PIN1 has also been displayed to stimulate prostate cancer cell proliferation and migration.^33^ Additionally, several studies have presented that PIN1 can stimulate Vimentin and N-cadherin protein expression and significantly reduce E-cadherin expression in cells, that is, PIN1 can promote EMT in cells. Nakada et al. found that PIN1 could activate STAT3 to induce EMT in gallbladder cancer cells,^34^ and Kim et al. also acquired the same finding in breast cancer.^35^ Shortly speaking, in the process of cancer development, PIN1 can regulate the biological function of cells by affecting complex signaling pathways. In this research, both miR-150-5p and PIN1 were highly expressed in HEp-2 cells, and the results suggested that high PIN1 expression could greatly promote the cell proliferation and invasion inhibited by high miR-150-5p expression.

## Conclusions

In summary, we have discovered that miR-150-5p can be implicated in the progression of LSCC and inhibit laryngeal carcinoma HEp-2 cells to proliferate and invasively spread for the first time. This RNA also has been found to prevent the HEp-2 cells from proliferating and invading by targeted regulation of PIN1. In short, this study provides a novel target for diagnosis and treatment of laryngeal cancer. Nevertheless, the role of miR-150-5p is complicated, so its mechanism still requires further exploration.

## Authors' contributions

Hailin Chen and Jianpiao Cai conceived and designed the experiments; Baowen Du and Zhengzheng Luo analyzed and interpreted the results of the experiments; Xuwei Cai drafted the manuscript. All authors approve the submitted manuscript.

## Funding

No funding source to declare.

## Data availability

The datasets used or analyzed during the current study are available from the corresponding author upon reasonable request.

## Conflicts of interest

The authors declare no conflicts of interest.
